# Metformin Inhibits Glutaminase Activity and Protects against Hepatic Encephalopathy

**DOI:** 10.1371/journal.pone.0049279

**Published:** 2012-11-15

**Authors:** Javier Ampuero, Isidora Ranchal, David Nuñez, María del Mar Díaz-Herrero, Marta Maraver, José Antonio del Campo, Ángela Rojas, Inés Camacho, Blanca Figueruela, Juan D. Bautista, Manuel Romero-Gómez

**Affiliations:** 1 Unit for Clinical Management of Digestive Diseases and CIBERehd, Hospital Universitario de Valme, University of Sevilla, Sevilla, Spain; 2 Department of Molecular Biology, University of Sevilla, Sevilla, Spain; Georgia Health Sciences University, United States of America

## Abstract

**Aim:**

To investigate the influence of metformin use on liver dysfunction and hepatic encephalopathy in a retrospective cohort of diabetic cirrhotic patients. To analyze the impact of metformin on glutaminase activity and ammonia production *in vitro*.

**Methods:**

Eighty-two cirrhotic patients with type 2 diabetes were included. Forty-one patients were classified as insulin sensitizers experienced (metformin) and 41 as controls (cirrhotic patients with type 2 diabetes mellitus without metformin treatment). Baseline analysis included: insulin, glucose, glucagon, leptin, adiponectin, TNFr2, AST, ALT. HOMA-IR was calculated. Baseline HE risk was calculated according to minimal hepatic encephalopathy, oral glutamine challenge and mutations in glutaminase gene. We performed an experimental study *in vitro* including an enzymatic activity assay where glutaminase inhibition was measured according to different metformin concentrations. In Caco2 cells, glutaminase activity inhibition was evaluated by ammonia production at 24, 48 and 72 hours after metformina treatment.

**Results:**

Hepatic encephalopathy was diagnosed during follow-up in 23.2% (19/82): 4.9% (2/41) in patients receiving metformin and 41.5% (17/41) in patients without metformin treatment (logRank 9.81; p = 0.002). In multivariate analysis, metformin use [H.R.11.4 (95% CI: 1.2–108.8); p = 0.034], age at diagnosis [H.R.1.12 (95% CI: 1.04–1.2); p = 0.002], female sex [H.R.10.4 (95% CI: 1.5–71.6); p = 0.017] and HE risk [H.R.21.3 (95% CI: 2.8–163.4); p = 0.003] were found independently associated with hepatic encephalopathy. In the enzymatic assay, glutaminase activity inhibition reached 68% with metformin 100 mM. In Caco2 cells, metformin (20 mM) decreased glutaminase activity up to 24% at 72 hours post-treatment (p<0.05).

**Conclusions:**

Metformin was found independently related to overt hepatic encephalopathy in patients with type 2 diabetes mellitus and high risk of hepatic encephalopathy. Metformin inhibits glutaminase activity *in vitro*. Therefore, metformin use seems to be protective against hepatic encephalopathy in diabetic cirrhotic patients.

## Introduction

Hepatic encephalopathy (HE) is one of the major complications of liver cirrhosis affecting one third of cirrhotic patients [1]. It has relevant socio-economic impact since HE reduces quality-of-life and is associated with higher mortality rate [2]. HE occurs as a result of the coexistence of hyperammonemia and inflammation in patients with liver dysfunction and/or porto-systemic shunts [3]. Ammonia production takes place mainly in the small intestine where glutaminase type K activity is crucial for the pathogenesis of HE [4]. Type 2 diabetes mellitus and insulin resistance (IR) are characterized by the release of pro-inflammatory cytokines, such as TNFα and IL-6, resulting in an inflammatory state [5]. Diabetes has been independently related to control of active variceal bleeding [6] and is associated with an increased risk of hepatocellular carcinoma development [7]. Type 2 diabetes mellitus has also been found associated with hepatic encephalopathy in patients with HCV-related cirrhosis [8]. Insulin sensitizers, like metformin, decrease insulin secretion and reduce hyperinsulinemic state. Metformin increases beta oxidation and reduces the hepatic gluconeogenesis via activation of AMP-K pathway; decreases intestinal glucose absorption and increases glucose uptake in skeletal muscle [9]. Recently, it has been found able to modulate the expression of cytokines, such as TNFα [10]. Thus, IR state could influence hepatic encephalopathy development in patients with cirrhosis. Insulin-sensitizers seem to decrease HCC in patients with cirrhosis C [11]. Therefore, the ammonia production, IR and the pro-inflammatory state seem to trigger cirrhosis progression, and may be interesting as therapeutic targets in the near future, improving the prognosis of cirrhotic patients.

**Table 1:** Comparison of baseline characteristics between groups.

	MET group (n = 41)	Non-MET group (n = 41)	Significance
**Age (years)**	60.2±9	60.4±10	0.908
**Sex, males**	34 (82.9%)	28 (68.3%)	0.123
**Child-Pugh score**	5.9±1.0	6.3±1.6	0.194
**MELD score**	9.0±2.4	9.9±4.2	0.285
**Etiology of cirrhosis**			0.476
**Alcohol**	26 (63.4%)	20 (48.8%)	
**HCV**	9 (22%)	13 (31.7%)	
**HBV**	1 (2.4%)	1 (2.4%)	
**Autoimmune**	0 (0%)	2 (4.9%)	
**Others**	5 (12.2%)	5 (12.2%)	
**HOMA-IR**	8.3±5.2	6.7±4.3	0.203
**Insulin (µU/mL)**	24.2±16.7	19.5±12.9	0.231
**Glucose (mmol/L)**	8.5±5.4	9.3±3.4	0.619
**Glucagon (pg/mL)**	101.2±45.5	111.9±66	0.547
**TNFr2 (pg/mL)**	14.3±8.9	18.2±7.7	0.203
**Leptin (ng/mL)**	20.2±16.6	20±16.9	0.977
**Adiponectin (µg/L)**	12.7±3.4	17.6±11.7	0.159
**AST (IU/L)**	55.1±63.8	44.3±25.8	0.553
**ALT (IU/L)**	43.6±43.9	45.3±45.3	0.905
**Ascites**	7 (17.1%)	18 (43.9%)	0.008
**Variceal bleeding**	5 (12.2%)	6 (14.6%)	0.746
**Follow-up (months)**	39.6±28.3	45.4±26.5	0.344

**Table 2:** Univariate analysis between hepatic encephalopathy and outcomes.

	Hepatic encephalopathy	Significance
**Metformin use**		0.002 (Log Rank 9.81)
**Yes**	4.9% (2/41)	
**No**	41.5% (17/41)	
**GLS gene alteration**		0.018 (Log Rank 5.57)
**Yes**	21.4% (6/28)	
**No**	45.4% (10/22)	
**Altered OGC & MHE**		0.006 (Log Rank 7.57)
**Yes**	45.8% (11/24)	
**No**	19.2% (5/26)	

GLS: glutaminase. OGC: oral glutamine challenge. MHE: minimal hepatic encephalopathy.

The aim of this study was to determine whether the metformin use was associated with decreased risk of hepatic encephalopathy in diabetic cirrhotic patients and to analyze the ability of metformin to inhibit glutaminase activity *in vitro*.

**Figure 1:**
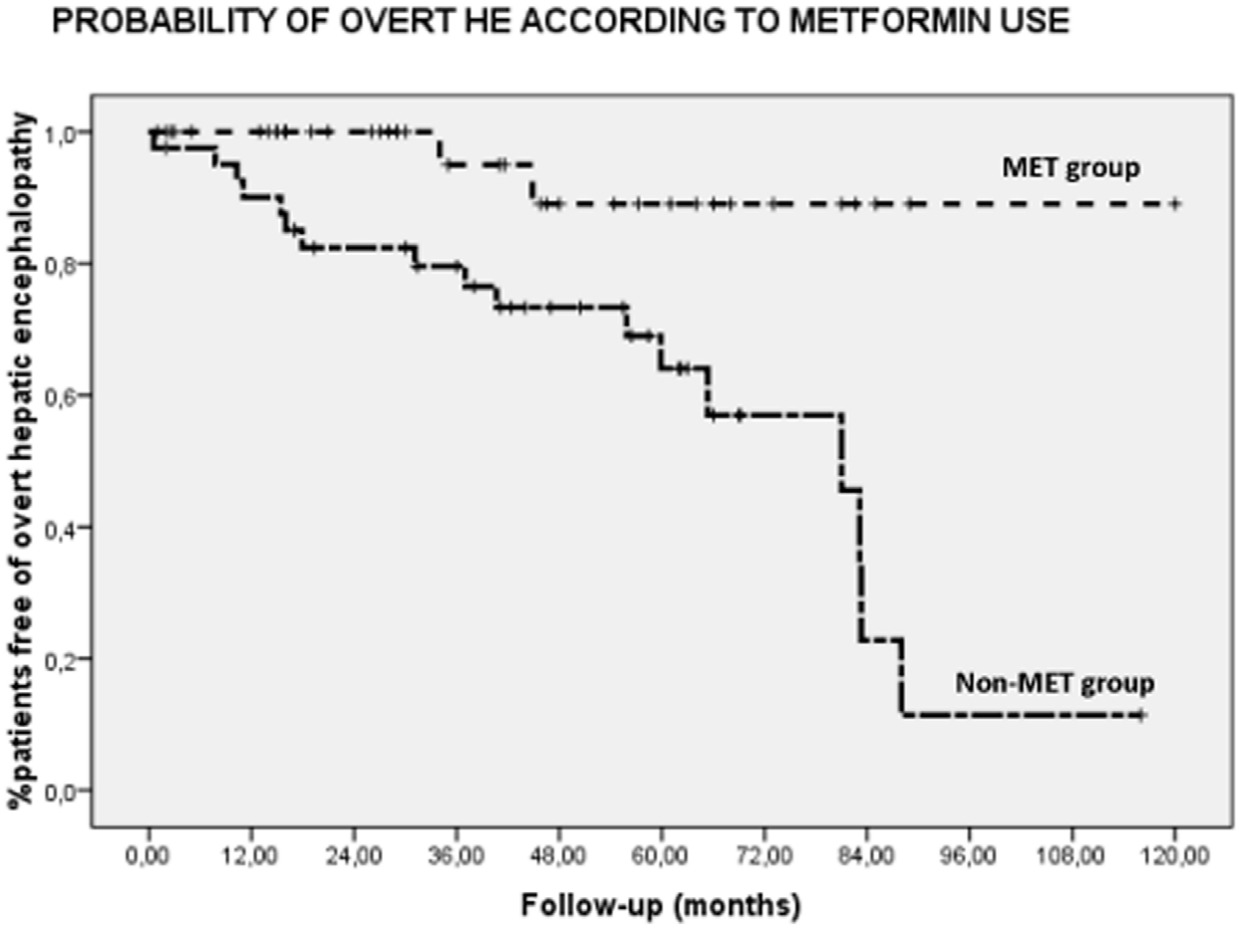
Kaplan Meier curve showing the impact of metformin use on hepatic encephalopathy (n = 82; log Rank: 9.45; p = 0.002).

## Methods

### Patients

Eighty-two consecutive diabetic cirrhotic patients from the Unit for Clinical Management of Digestive Diseases, University Hospital of Valme, were included. The study started either with the first visit to Hepatology office or with the first hospital admission and outcomes to finish were survival and liver transplantation. Exclusion criteria were: age≤18 years; non-diabetic patients; patients with type 1 diabetes mellitus; and patients with treatment ongoing for cirrhosis. The protocol was approved by the CEIC of University Hospital of Valme (Sevilla, Spain) and all patients provided written informed consent to participate in this study. The study was conducted in accordance with the ethical guidelines of the Declaration of Helsinki and International Conference on Harmonization Guidelines for Good Clinical Practice. A total of 41 cases and 41 controls were included. They were classified according to insulin sensitizers experienced. Cases were defined as patients who underwent metformin treatment, while controls were defined as cirrhotic patients with type 2 diabetes mellitus without metformin treatment. Metformin-experienced average time was 33.4±26.7 months. Type 2 diabetes mellitus was diagnosed according to the *American Diabetes Association* [12].

**Table 3:** Multivariate analysis according to overt HE.

Hepatic encephalopathy	Multivariate
**Metformin use**	[H.R. 11.4 (95% CI: 1.2–108.8); p = 0.034]
**Age at diagnosis**	[H.R. 1.12 (95% CI: 1.04–1.2); p = 0.002]
**Female sex**	[H.R. 10.4 (95% CI: 1.5–71.6); p = 0.017]
**HE risk**	[H.R. 21.3 (95% CI: 2.8–163.4); p = 0.003]

### Biochemical and Clinical Parameters

Baseline analysis, using commercial tests, included: insulin, glucose, glucagon, TNFr2, leptin, adiponectin, AST and ALT. HOMA-IR was calculated [glucose (mmol/L) * Insulin (IU/ml)/22,5]. Cirrhosis was defined and based on liver biopsy, ultrasound, endoscopic analysis and biochemical parameters.

**Figure 2:**
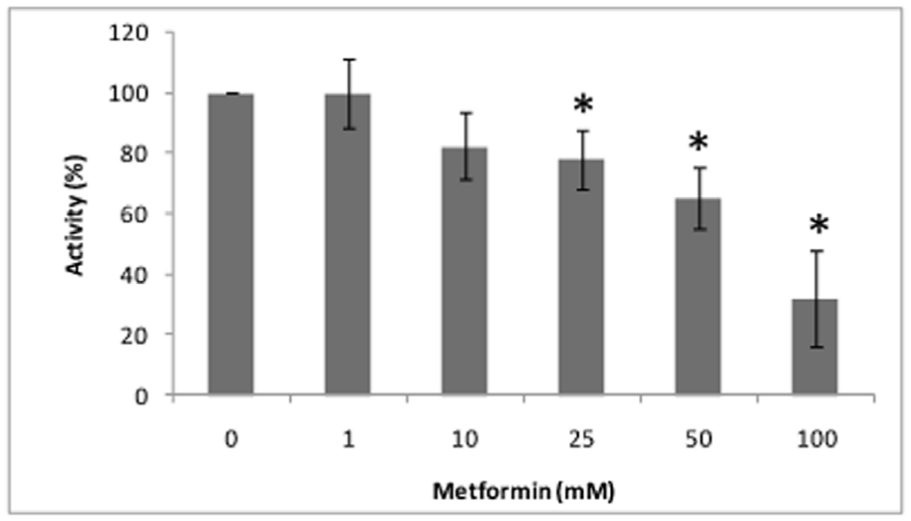
Glutaminase activity in chemical assay (%), according to metformin concentration. Each bar represents the mean±SD (all experiments were conducted by triplicate).

**Figure 3:**
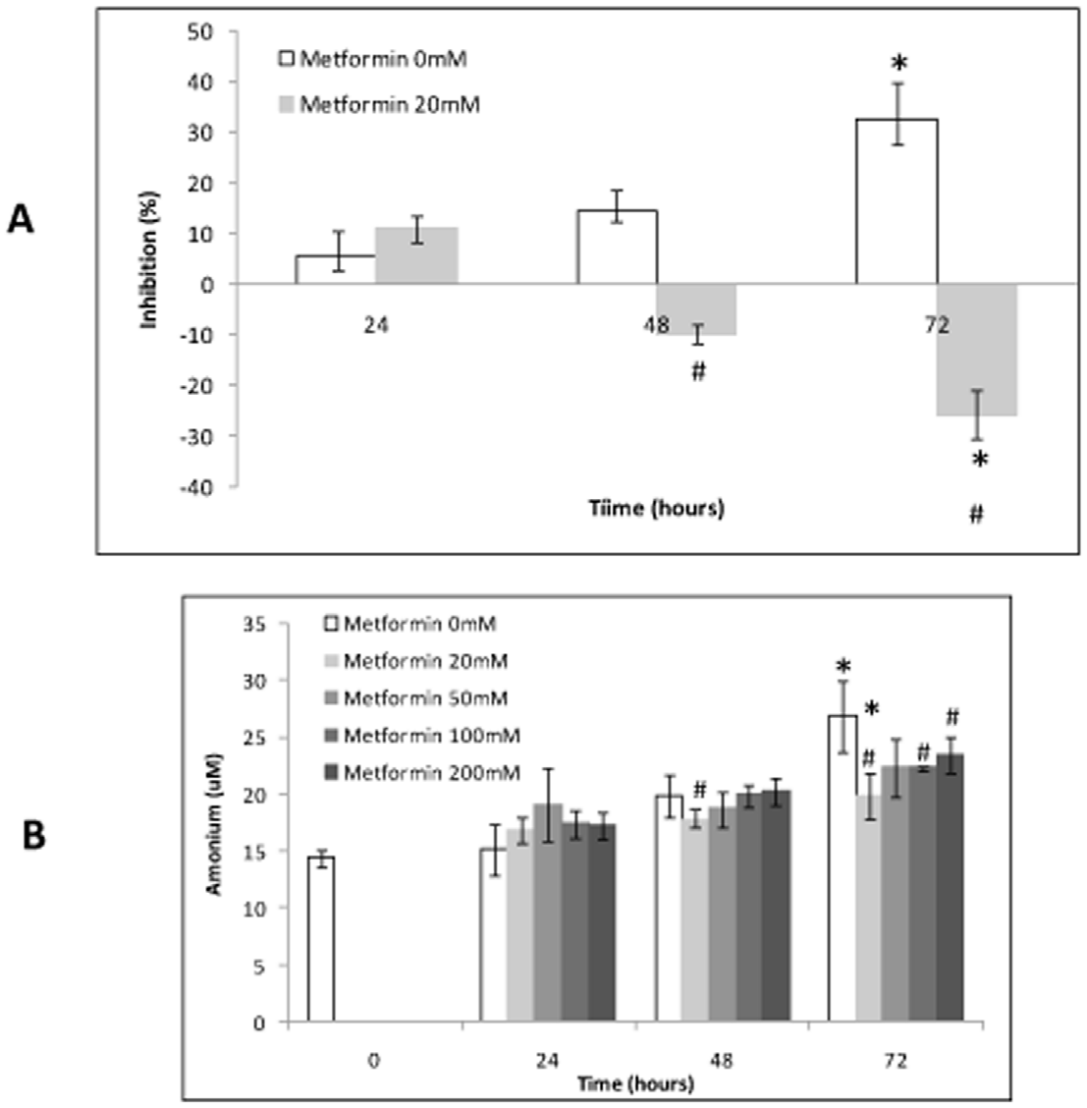
Effect of metformin on glutaminase activity *in vitro.* 3A) Glutaminase activity inhibition in cells assay (%), according to metformin concentration; 3B) Ammonia concentration in cells assay, according to metformin concentration. Each bar represents the mean ± SD (all experiments were conducted by triplicate). *p≤0.05 vs. the corresponding control sample. #p≤0.05 vs. the same group collected at the previous time point.

### Encephalopathy Management

Minimal hepatic encephalopathy (MHE) was diagnosed based on psychometric hepatic encephalopathy score (PHES) and critical flicker frequency (CFF) (Hepatonorm™ Analyzer (R&R Medi-Business Freiburg GmbH, Freiburg, Germany)). This battery comprises the digit symbol test (DST), the number connection test A (NCT-A), the number connection test B (NCT-B), the serial dotting test (SDT), and the line drawing test (LDT). Patients were classified as having MHE when the PHES score was less than −4 points or the CFF value was below the cut-off (38 Hz) [13]. For oral glutamine challenge (OGC) analysis, blood samples were taken at baseline and 60 minutes following glutamine load (10 g glutamine dissolved in 100 ml water (L-Glutamine, SHS S.A., Spain)). Ammonia was measured using the DaFonseca-Whollheim method in an auto-analyzer (Hitachi 911; Roche Diagnostics, Mannheim, Germany). A pathological response curve for glutamine tolerance was defined as an ammonia rise to >128 µg/dL at 60 minutes after the glutamine intake [14]. Genetic studies included length of microsatellites in the 5′UTR region of glutaminase gene together with haplotype TACC, as previously described [15]. Baseline high risk for hepatic encephalopathy was defined according to MHE, altered OGC and genetic alterations. Patients with MHE (PHES<−4 or CFF<38 Hz) and altered OGC (NH3>128 mg/dl at 60 minutes) or showing genetic (Large/large microsatellite or non-TACC haplotype) were classified as high risk of HE development (48%; 24/50) and the rest (52%; 26/50) as low risk of overt HE.

### Experimental Study

We performed an experimental study *in vitro* (chemical assay and cells assay) to investigate the glutaminase activity inhibition, according to different metformin concentrations.

First, in an enzymatic assay, we tested different metformin doses (0, 10, 25, 50 and 100 µM) with a constant glutamine concentration (100 mM). Ammonia production was measured to determine the glutaminase activity.

On the other hand, human colonic epithelial mammalian cell line of Caco2 (American Type Culture Collection, ATCC) was maintained in DMEM medium pH 7.4 supplemented with 10% fetal bovine serum, 2.2 g/L HCO_3_Na, 100 mM sodium pyruvate, 0.292 gr/L glutamine, 100 U/mL penicillin, 100 µg/mL streptomycin and 0.25 µg/mL amphotericin in 5% CO_2_ at 37°C. Cell assay was initiated 24 hr after seeding. Caco2 cells (10000 cells/cm^2^) were cultured in presence of different metformin doses (0, 20, 50, 100 and 200 mM) and samples (cell pellet and cultured medium) were collected after 0, 24, 48 and 72 hr post-treatment. Glutaminase activity was determined by the measurement of ammonia production.

### Statistical Analysis

Results are expressed as mean±SD of 3 independent experiments. Data were compared using ANOVA with the Least Significant Difference (LSD) test as posthoc multiple comparison analysis. We used the Kaplan-Meier method (log rank test to compare curves), Chi-square and T-student. Cox’s regression was used for univariate analysis and those variables with p<0.150 were entered into the multivariate analysis. The statistical differences were placed at p≤0.05.

## Results

### Effect of Metformin Use on Hepatic Encephalopathy

Baseline epidemiological, biochemical and liver function test from both groups of patients are shown in **Table 1**. No differences were found in sex, age, etiology of cirrhosis and liver function (including Child-Pugh score and MELD). The etiology of cirrhosis was alcoholic cirrhosis (n = 46; 56.1%), HCV-related (n = 22; 26.8%), HBV-related (n = 2; 2.4%), cryptogenic (n = 10; 12.3%) or autoimmune (n = 2; 2.4%). Gender distribution was 75.6% men (62/82) and 24.4% females (20/82), with mean age of 60.3±9.5 years. Liver function according to Child-Pugh stage was: 57 patients (69.5%) at Child-Pugh Stage A; 24 patients (29.3%) at Stage B and 1 patient (1.2%) at stage C. Mean Child-Pugh score was 6.1±1.4 and MELD score was 9.5±3.5. Average follow-up was 42.5±27.4 months. Nineteen patients (23.2%) developed episodes of overt HE during follow-up. These bouts were related to diuretics (31.6%; 6/19), variceal bleeding (26.2%; 5/19) and infections (15.8%; 3/19), being 26.4% (5/19) spontaneous. Univariate analysis demonstrated altered Child-Pugh, OGC, PHES, CFF, genetic factors and metformin use were associated with the risk of overt hepatic encephalopathy (**Table 2**). In the metformin group, we found 4.9% of cases (2/41), while 41.5% (17/41) occurred in controls (log Rank 9.81; p = 0.002) (**Fig. 1**). In multivariate analysis, metformin use [H.R. 11.4 (95% CI: 1.2–108.8); p = 0.034], age at diagnosis [H.R. 1.12 (95% CI: 1.04–1.2); p = 0.002], female sex [H.R. 10.4 (95% CI: 1.5–71.6); p = 0.017] and HE risk [H.R. 21.3 (95% CI: 2.8–163.4); p = 0.003] were found independently associated with EH (**Table 3**). On the other hand, overall survival rate reached a trend in cirrhotic patients metformin-experienced: 92.7% (38/41) of MET group survived and 82.9% (34/41) of controls.

### Effect of Metformin on High Risk Patients

Patients with MHE (PHES<−4 or CFF<38 Hz) and altered OGC (NH3>128 mg/dl at 60 minutes) or showing genetic profile (Large/large microsatellite or non-TACC haplotype) were classified as high risk of HE development. Metformin use, in these cohorts, was associated with lower HE bouts, both in high-risk and low risk patients (log Rank 7.57; p = 0.006).

### Effect of Metformin on Glutaminase Activity *in vitro*

In chemical assay, 17.5% of glutaminase activity inhibition was obtained with a metformin concentration of 10 mM and up to 68% inhibition was reached using 100 mM. Therefore, a dose-dependent glutaminase activity was observed with metformin use (**Fig. 2**). In Caco2 cells, 20 mM of metformin showed 24% inhibition of glutaminase activity at 72 hours compared with the control at the same time (p<0.05) (ammonia production was decreased from 26.85±0.74 µM to 19.9±2.05 µM; p<0.05) (**Fig. 3A**). Other metformin concentrations (50, 100 and 200 mM) inhibited also the glutaminase activity, but this effect was lower than 20 mM of metformin, as reflected by the presence of ammonium in cultured medium (**Fig. 3B**).

## Discussion

The major findings from this work are: first, in the experimental study, we obtained a partial inhibition of glutaminase activity (about 20%), both in the chemical and cells assays when compared with control experiments. Glutaminase converts glutamine in glutamic acid, which is indispensable for cell function, together with ammonia and free radicals. Therefore, this partial inhibition is probably enough to prevent complications in cirrhotic patients (in particular hepatic encephalopathy), preserving the beneficial effects of glutaminase. Second, we observed an eight-fold lower risk of hepatic encephalopathy in metformin-experienced patients (4.9% vs 41.5%; p = 0.002), despite both cohort were similar in liver function and HE risk score. Metformin effects on glutaminase activity and inflammatory state (modulated by glycemic control) could explain, at least in part, this result. HOMA-IR correlates with protein-C-reactive activity and patients receiving metformin showed a trend to lowering TNFr2 levels than non-metformin treated patients (data not shown). Interestingly, in Child-Pugh A patients, HOMA index was independently associated with higher rate of overt HE, supporting the hypothesis that insulin resistance syndrome could promote inflammation and increased risk of overt HE. Indeed, fecal calprotectin correlated with critical flicker frequency and HE grading [16].

Type 2 diabetes mellitus has been found associated with hepatic encephalopathy in patients with Hepatitis C-related cirrhosis. The mechanisms by which diabetes could promote hepatic encephalopathy includes: a) inflammation states in cirrhotic patients has been associated with bacterial translocation, hepatic encephalopathy and risk of spontaneous bacterial peritonitis. Insulin resistance syndrome and type 2 diabetes mellitus are considered as an inflammatory state due to increased production of pro-inflammatory cytokines, such as TNFα and IL-6 [17]; b) motility impairment has been described in diabetic patients showing delayed duodenum-cecal transit time. It could promote small intestine bacterial overgrowth (SIBO) raising bacterial translocation rate. Indeed, SIBO was found in more than 60% of cirrhotic patients and it was strongly related to bacterial translocation [18]. Moreover, lactulose breath test was found altered in 8 out of 9 patients with previous bouts of hepatic encephalopathy; c) type 2 diabetes seems to play a role modulating several isoforms of glutaminase (GA). Three glutaminase isoforms have been described; kidney-type (KGA), liver-type (LGA) and type C (CGA). Baglietto-Vargas et al. demonstrated that KGA and LGA are present in endocrine pancreas (KGA in alpha cells and periphery of the islets and LGA in beta cells) and could have some role in the secretion of insulin [19]. In addition, type 2 diabetes promotes renal uptake of plasma glutamine for the production of urinary ammonia, activating KGA. Besides, streptozotocin-induced diabetic rats have demonstrated that hepatocytes use glutamine more rapidly than do hepatocytes from normal rats; as a consequence of that, glutaminase activity in diabetic rats is increased leading to a higher glutamine uptake and ammonia production. Furthermore, Watford et al. observed the increase in glutaminase activity in the small intestine in type 2 diabetes rats [20].

The effect of metformin on hepatic encephalopathy was stronger than expected. In spite of all these data support an active effect of metformin on cirrhotics, a selection bias could not be excluded in a retrospective analysis. Moreover, although metformin seems to be safer than exogenous insulin preventing cirrhosis complications, it may be difficult to maintain adequate blood glucose levels with insulin sensitizers alone. Thus, a balance between glucose control to avoid diabetes progression and insulin sensitivity to avoid cirrhosis complications is required. Other cirrhosis outcomes, particularly ascites, were also modified by metformin use (probably due to decrease inflammation [21]) but in a different manner and were beyond the aim of our study.

In conclusion, our results indicated that metformin use reduced the risk of hepatic encephalopathy in diabetic cirrhotic patients, probably by two mechanisms: inhibiting partially glutaminase activity and improving insulin sensitivity. A randomized control trial is warranted to confirm or not these data defining the usefulness of metformin in the management of liver cirrhosis.
